# Packaging biological cargoes in mesoporous materials: opportunities for drug delivery

**DOI:** 10.1517/17425247.2014.938636

**Published:** 2014-07-12

**Authors:** Justin Siefker, Pankaj Karande, Marc-Olivier Coppens

**Affiliations:** ^a^ University College London, Department of Chemical Engineering and EPSRC Frontier Engineering Centre for Nature Inspired Engineering, Torrington Place, London, WC1E 7JE, UK; ^b^ Rensselaer Polytechnic Institute, Department of Chemical and Biological Engineering and Center for Biotechnology and Interdisciplinary Studies, 110 Eighth Street, Troy, NY 12180, USA+1 518 276 4459; karanp@rpi.edu; ^c^ University College London, Department of Chemical Engineering and EPSRC Frontier Engineering Centre for Nature Inspired Engineering, Torrington Place, London, WC1E 7JE, UK+44 20 7679 7369; +44 20 7383 2348; m.coppens@ucl.ac.uk

**Keywords:** compartmentalization, confinement, controlled release, drug delivery, extra-particle effects, intra-particle effects, MCM-41, mesoporous silica, nanoparticle therapeutics, protein therapeutics, SBA-15, targeted delivery

## Abstract

***Introduction:*** Confinement of biomolecules in structured nanoporous materials offers several desirable features ranging from chemical and thermal stability, to resistance to degradation from the external environment. A new generation of mesoporous materials presents exciting new possibilities for the formulation and controlled release of biological agents. Such materials address niche applications in enteral and parenteral delivery of biologics, such as peptides, polypeptides, enzymes and proteins for use as therapeutics, imaging agents, biosensors, and adjuvants.

***Areas covered:*** Mesoporous silica Santa Barbara Amorphous-15 (SBA-15), with its unique, tunable pore diameter, and easily functionalized surface, provides a representative example of this new generation of materials. Here, we review recent advances in the design and synthesis of nanostructured mesoporous materials, focusing on SBA-15, and highlight opportunities for the delivery of biological agents to various organ and tissue compartments.

***Expert opinion:*** The SBA-15 platform provides a delivery carrier that is inherently separated from the active biologic due to distinct intra and extra-particle environments. This permits the SBA-15 platform to not require direct modification of the active biological therapeutic. Additionally, this makes the platform universal and allows for its application independent of the desired methods of discovery and development. The SBA-15 platform also directly addresses issues of targeted delivery and controlled release, although future challenges in the implementation of this platform reside in particle design, biocompatibility, and the tunability of the internal and external material properties. Examples illustrating the flexibility in the application of the SBA-15 platform are also discussed.

## 1. Introduction

The last few decades have brought significant advances in the design and synthesis of novel drug delivery systems [1-4]. These advances have focused on addressing critical challenges in improving systemic stability of drugs; designing better formulation strategies for drugs; achieving cell-, tissue- or organ-specific targeting; and providing controlled release of the active drug at the site of interest. These advances have collectively contributed to improved delivery systems and superior clinical outcomes in patients [5,6].

A systematic trend in drug development during the last two decades is the shift towards biologics [7]. Biologics comprise a broad range of molecules that are typically large molecular weight drugs, which include peptides, polypeptides, proteins, antibodies and enzymes. Biologics provide improved therapeutic end points when compared with small-molecule drugs in terms of efficacy, selectivity and targeting. However, they are limited by poor systemic and oral stability, are prone to proteolytic degradation and show high susceptibility to temperature and chemical agents [8]. Therefore, enhancing the stability and bioavailability of biologics in the physiological environment is critical for their therapeutic success.

Recent studies [9], including those from our group [10], demonstrate that confinement of biomolecules in nanostructured porous materials, with uniform pores slightly wider than the biomolecule, can significantly enhance molecular stability to proteases, temperature and solution conditions. Although this work bears certain similarities to prior work that has evaluated the effects of immobilisation on non-porous nanoparticles [11], confinement of biomolecules in nanoporous materials is fundamentally different. The key difference that changes the fundamental behaviour between confinement on the internal surface of porous materials and external surface adsorption is that porous, nanostructured materials inherently provide separate internal and external environments. This is due to the physical differences between the environments, including solvent effects, and surface proximity and chemistry. It follows that the interactions of the confined biomolecules with their environment are also inherently distinct. This affords greater design flexibility by decoupling considerations for the biomolecular cargo and the physiological environment. For this reason, many porous materials are currently being investigated for drug delivery applications, such as zeolites [12], mesoporous carbons [13], mesoporous silicas [14,15], metal organic frameworks [16,17], hydrogels [18], micelles [3] and calcium phosphate cements [19]. Although many of these materials show great promise, they also tend to either be sensitive to the external environment as a whole or are difficult to engineer internally. Material sensitivity to the environment can range from pH and ionic strength considerations, to general chemical instability and enzymatically mediated decomposition. Difficulties of internal engineering can be due to the dependence of internal properties on the synthetic steps required for tuning of the external properties or the lack of ability to tune the internal surface chemistry, porosity or tortuosity. For these reasons, many of these materials are not necessarily the best candidates for drug delivery. In contrast, a relatively new class of ordered mesoporous silica particles provides a promising framework that has broad flexibility in its chemical and structural features at multiple (∼ 2 nm to 1 µm) length scales. In this *expert opinion*, we focus on recent advances regarding a compelling mesoporous silica candidate that fits this niche, Santa Barbara Amorphous-15 (SBA-15) [20].

## 2. Current limitations of nanomaterials in drug delivery

Before evaluating any novel nanomaterial candidate for drug delivery, it is important to first evaluate where current materials fall short. Key properties that are desired in a nanomaterial candidate include selective compartmental distribution in organs and tissues, particle size uniformity, selective affinity-based interactions, material stability and biodegradability, versatile surface chemistry and tunable particle geometry [21]. For example, the small size (20 – 100 nm) of nanoparticles provides the perfect advantage due to enhanced permeability and retention (EPR) [22] in tumours because of increased porosity of the adjacent vasculature. This leads to non-specific, but selective biodistribution in tumours [23]. EPR has shown promise for drug delivery since the 1980s, but is still significantly limited due to heterogeneity of the effect in different tumours [23]. EPR is only one example of how the control of particle size is advantageous as it relies on the link between particle size, therapeutic effect and mechanism of action. However, as the mechanism of EPR is governed primarily by one parameter, cut-off size, this severely limits its application as a method of drug delivery. This illustrates the need for the next key property, particle size uniformity, which is also important in the specific targeting of tumorous tissues. Fenestrations in the vascular endothelium of pathological tissues allow for the easy transport of nanoparticles to the desired tissue, although this is complicated by the fact that fenestrations vary greatly in size, from ∼ 1 nm to 1.4 μm, depending on the tissue [24]. If the size of the therapeutic nanoparticle can be tuned to that of the fenestrations in the targeted tissue, then the nanoparticle may be guided to the desired tumour cells. Furthermore, the size and shape of a nanoparticle can dramatically change how it interacts with the physiological environment by limiting or mediating transport across membranes, and regulating persistence in circulation or clearance by the immune system [25-27]. With this in mind, if particle size and shape uniformity cannot be ensured, neither can the specificity of the nanoparticle’s interactions. Charge provides another design variable that can change the behaviour of nanoparticles in physiological environments. Charge can be used to promote specific and desirable interactions. For example, cationic nanoparticles have a higher internalisation rate due to their electrostatic attraction with the negative charge of cell membrane lipids [28]. However, electrostatic charge can also promote non-specific interactions and lead to non-specific distribution of particles, in addition to targeted tissues [29]. As such, the ideal charge for a particle depends on the desired application. Although no steadfast rules for the desired particle charge apply, fewer non-specific electrostatic interactions occur when the surface charge of a particle is lower.

Due to shortcomings of some synthetic nanoparticles, many researchers are also investigating the use of nanoparticles synthesised of biological materials. These materials usually consist of DNA [30], RNA [31], proteins [32], lipids [33] and carbohydrates [34,35]. Such materials can provide several advantages such as a high-packing density, which can lead to enhanced dosing, an increased flexibility of design as these materials naturally interact biologically and a lack of void volume as whole particles can be designed to be active. However, such nanoparticles have their own set of complications [36,37]. These complications can include the requirement for additives (such as trehalose) [38] and for conjugation or modification unique to the specific nanoparticle [39]. In addition, biological machinery is more apt to lead to undesired biological responses and possible instability [40]. This is due to the physiological familiarity of these materials, which can facilitate immunogenic response, lead to material degradation or unexpectedly initiate signalling pathways. This is well illustrated by the two major hurdles of siRNA therapeutic candidates: immunogenic response stimulated by the activation of an undesired signalling pathway, and degradation in serum due to the presence of ribonucleases [41].

As described previously, it is difficult to tune the specific properties of many nanoparticles under development in a manner that facilitates the desired biological response. Changing one desired property might change another one that is equally desirable for an application [42]. For example, altering the synthesis procedure to tune the interactions of a hydrogel with the physiological environment is likely to additionally modulate the structural properties of the hydrogel and the therapeutic–hydrogel interactions. This could result in significant attenuation of desired therapeutic–hydrogel interactions. Another example would be the chemical functionalisation of liposome-based particles. Although functional groups can be added, they must not significantly disrupt the defining lipid bilayer and must follow the general rules of partitioning between the hydrophilic and lipophilic phases. For nanoparticles with desirable material properties, one approach to address this shortcoming is to decouple particle–therapeutic and particle–physiological interactions. This separation can be achieved through the use of porous materials with properties that can be fine-tuned with near-atomic resolution [43], such as SBA-15. We will review these materials in greater detail subsequently in this review.

## 3. A compelling nanoparticle candidate

An ideal nanoporous material for drug delivery would address many of the challenges highlighted previously. Porous materials with biomolecules confined in the pores allow for separation of particle–biomolecule and particle–physiological interactions to the interior and exterior of a particle, respectively. The pore sizes that allow for the separation of these interactions for protein biologics are typically in the mesoporous range (2 – 50 nm pore diameter). Challenges of engineering the interface between the internal pore environment and the biomolecule include issues such as proteolysis, pH induced aggregation, shear induced denaturing, hydrophobic denaturing and the regulation of biomolecular diffusion [44]. Although protection from proteolytic cleavage and pH-induced aggregation is essential for almost all biological therapeutics, it is particularly important for therapeutics that are orally administered due to the high presence of proteases and the variability of pH (1.5 – 6.5) in the gastrointestinal tract [45,46]. The combination of these two issues is one of the major hurdles for oral delivery in biological therapeutics.

As the use of porous materials isolates particle–therapeutic interactions to the interior of the particle, the extra particle challenges deal primarily with the physiological interactions of the particle and global targeting of the therapeutic. Extra particle challenges include targeted delivery of the therapeutic, isolation of particles, crossing of desired barriers and mitigation of non-discriminant interactions with proteins and biomolecules in serum and other biological fluids. It is essential for each of these challenges to be addressed to localise the therapeutic effect, minimise toxicity issues and achieve effective therapeutic delivery.

There are several classes of porous nanoparticles currently being investigated to address challenges in therapeutic delivery. These include polymeric gels [47], mesoporous carbon [48], and mesoporous silica [49]. Each type of these materials has its advantages and disadvantages. It is easy to synthesise and control the degradation of polymeric gels, but the pore size distribution and pore environment are difficult to control [42]. Mesoporous carbons and silicas have well-characterised synthesis methods and chemistry, but form persistent rigid structures in physiological conditions [50,51]. Although this ensures particle stability, it also makes particle clearance and accumulation a significant concern that must be addressed during the therapeutic design phase. Out of the possible classes of porous nanoparticles that would be well suited for biological therapeutic delivery, mesoporous silica SBA-15 presents a compelling candidate.

## 4. Nanoparticle candidate SBA-15

SBA-15 is a mesoporous silica in which the pores are templated by tri-block copolymer micelles [20]. The material contains hexagonally ordered cylindrical mesopores in one dimension, ranging from 5 to 15 nm in diameter with a very narrow pore size distribution (< 2 nm fwhm) [52]. The surface is hydrophilic and weakly negatively charged (< -0.08 C/m^2^) [53] under physiological conditions. The internal and external surfaces of SBA-15 provide easily accessible silanol groups that can be functionalised through the use of silane chemistry, thereby providing an avenue to modify the surface chemistry.

SBA-15 is synthesised by first dissolving Pluronic 123 (P123) tri-block copolymer (EO_20_PO_70_EO_20_, where EO is ethylene oxide and PO is propylene oxide) in an acidic aqueous environment. Acidic, high-ionic strength P123 micelles transform from spherical to cylindrical when tetraethyl orthosilicate is added to the solution [54,55], which hydrolyses into silica precursors and ethanol; the released ethanol increases the rate of self-assembly of the cylindrical micelles and the polymerising silica over the course of several hours, gradually leading up to a composite consisting of hexagonally ordered cylindrical micelles of P123, surrounded by a continuous silica phase. This transformation continues via hydrothermal aging in an oven. The temperature of the hydrothermal aging determines the diameter of the cylindrical micelles, and, ultimately, the pore diameter. Depending on the process conditions (stirring rate, presence of inorganic salts, pH, temperature, etc.), materials in a variety of shapes can be produced, such as particles, fibres and films [56-59]. For example, minimal stirring promotes the formation of monodispersed, approximately micron-sized rods, with a width of a few hundred nanometres [60,61].

Subsequently, samples are washed, dried and the P123 template is removed by either calcination or solvent extraction [52,61]. If calcination is used to remove the template, sonication is often used to disperse the particles homogenously in solution prior to further processing [62]. The two main avenues to functionalise the surface of SBA-15 are surface grafting and co-condensation. Surface grafting is very attractive due to chemical flexibility and ability for complete coverage of the functionalised surface [43]. On the other hand, co-condensation is completed during the hydrolysis step of the synthesis; hence, it can interfere with the structural integrity of the synthesised particles. Regardless of the functionalisation method implemented, Fourier transform infrared spectroscopy (FTIR) and nuclear magnetic resonance spectroscopy, among other spectroscopic tools, can be used to analyse the surface chemistry.

**Figure 1.**
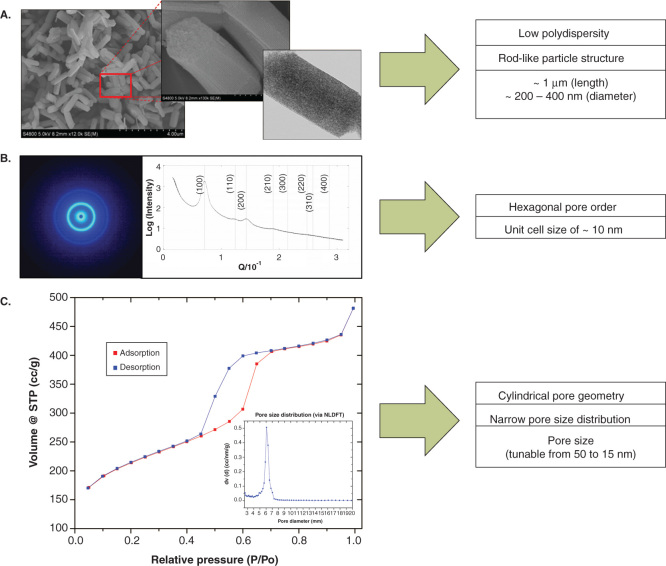
**Characterisation of SBA-15.** Illustration of a typical combination of methods used for the characterisation of SBA-15. The key results of each analysis are included with each example. (**A**) FE-SEM and HRTEM (far right), (**B**) SAXS, (**C**) N_2_ adsorption/desorption analysis.

FE-SEM: Field emission scanning electron microscopy; HRTEM: High-resolution transmission electron microscopy; SBA-15: Santa Barbara Amorphous-15; SAXS: Small-angle x-ray scattering.

Typical characterisation of SBA-15 includes a combination of analysis methods that characterise both the particle and the pore structure [20]. These methods include gas sorption analysis, x-ray diffraction or small angle x-ray scattering (SAXS), and imaging via field emission scanning and high-resolution transmission electron microscopy (FE-SEM and HRTEM) [10]. An example is shown in Figure 1. The nitrogen adsorption analysis shows a narrow pore size distribution around an average pore size of 5.9 nm. The SAXS peaks are characteristic for a hexagonally ordered array of pores with a cell unit size of 9.9 nm. The FE-SEM and HRTEM analyses show a mixture of homogenous cylindrical particles ∼ 1 µm in length and 400 nm in diameter. Apart from the mesopores, SBA-15 might contain micropores in the pore walls (< 2 nm wide) [63].

SBA-15 could address many of the shortcomings of current nanoparticle candidates. The key attributes that make SBA-15 a compelling nanoparticle candidate for therapeutic delivery can be classified into two categories: the external surface or extra-particle properties, and the mesopore or intra-particle properties. As the surface of SBA-15 is easily functionalised, selective ligand-based interactions can be mediated and targeting groups can be attached [43,64]. Particle size and shape uniformity can also be addressed by properly tuning the SBA-15 synthesis procedure to ensure that the particles interact uniformly with the environment. Shapes such as rods, spheres and platelet-like geometries can be selected to mediate the desired physiological response [57,59]. Undesired interactions due to high-surface charge, as observed in many other candidate materials, are addressed by the naturally weak electrostatic surface charge of the SBA-15 particles, which can be further modulated with surface functionalisation [53,43]. The covalent bonds of the SBA-15 framework are stable, ensuring the persistence of the particles under physiological conditions [51]. However, it is essential to note that after incubation in simulated physiological fluid for 60 days, silica lixiviation from SBA-15 does occur and results in partial loss of mesostructure. The loss of mesostructure due to lixiviation, however, can be significantly reduced by surface functionalisation. All of the basic geometric properties of SBA-15 can be tuned to varying degrees during the synthesis process [57,58], and the surface chemistry can be completely customised using silane chemistry [43]. The particles have a rigid structure that protects the integrity of the internal porosity and can also be independently functionalised, separately from the internal pore surface. This allows for the adjustment of external surface properties to address issues of targeted delivery and biocompatibility, such as the reduction of particle aggregation in physiological environments.

**Figure 2.**
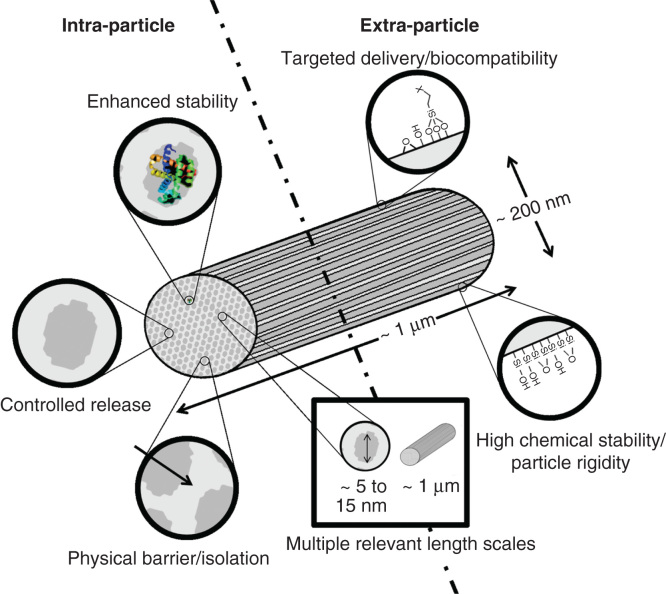
**Intra- and extra-particle aspects of SBA-15.** Design aspects of SBA-15 are illustrated, highlighting their possible application to address intra- and extra-particle challenges.

Internally, the properties of the SBA-15 mesopores govern the particle–therapeutic interactions. The narrow, uniformly sized mesopores ensure that all of the protein cargo interacts identically with the pore surface. In addition, the protein cargo is only exposed to a constant, concave curvature, which has been shown to have dramatic stabilising effects [10]. The pores also provide direct physical protection from external hazards such as proteases due to the rigid structure of SBA-15. The weakly charged internal surface can be selectively functionalised, independently from the external surface, allowing for controlled release and enhanced stability of protein cargo. Figure 2 presents an overview of how SBA-15 can address the intra- and extra-particle design constraints.

SBA-15: Santa Barbara Amorphous-15.

## 5. SBA-15 as a carrier of biological therapeutics

Current research on the use of SBA-15 for therapeutics has followed two main avenues: the fundamental understanding and handling of the material, and specific applications, primarily using non-biological therapeutics. Although the use of SBA-15 for non-biological therapeutics is beyond the scope of this article, some of the research will be reviewed as it demonstrates methods and possible applications that could be expanded to biological therapeutics. First, we will discuss fundamental aspects of pore curvature and isolation effects [10]. Then, we will transition to the application of material tuning and functionalisation to affect biological interactions [43,64], and controlled release [65,66].

As discussed previously, SBA-15 contains hexagonally ordered cylindrical mesopores with a narrow pore size distribution. Compared with most other mesoporous silicas, this gives it the unique ability to expose the biological cargo to only concave curvature upon adsorption. This property of SBA-15 was recently investigated by Sang *et al*. using myoglobin and lysozyme as model proteins [10]. Sang *et al*. adsorbed the model proteins onto SBA-15 of varying pore diameter with both propylated and non-functionalised surface chemistries, and then quantified the changes in catalytic activity and secondary structure (via FTIR). The authors found that the model proteins adsorbed on the propylated surface were less catalytically active and had significant changes to their secondary structure when compared with the same proteins free in solution. The changes in secondary structure showed no dependence on the diameter of the pore. These results suggest that a phase inversion and denaturing occurs on a hydrophobic interface, as one might suspect [10], and that pore curvature does not play a dominant role in this system. In contrast, the model proteins adsorbed on the non-functionalised, hydrophilic surface show extraordinary increases in activity, and a secondary structural dependence on pore diameter. As the pore diameter decreases to approximately that of the size of the model proteins, the secondary structure of the proteins approach that of the free protein in solution. This trend provides insight into the fundamental behaviour of confined protein and demonstrates that pore curvature can play a significant role in porous environments.

In trying to understand why this effect is so dramatic, one can look at a structural analogue found in nature, the GroEL/ES chaperonin complex. The GroEL/ES chaperonin complex is required to finish the proper folding and for the refolding of some misfolded bacterial proteins [67]. The complex uses ATP to partially unfold or destabilise a protein, and then facilitates the proper refolding via a kinetically mediated minimisation of free energy [67]. The relevance of this example becomes much more apparent once the structure of SBA-15 and the GroEL/ES chaperonin complex are compared. Both structures confine a protein in a hydrophilic, cylindrical pore of approximately the same size as the protein in which the surface is negatively charged [67]. This suggests that there is something intrinsically fundamental to the confinement and curvature effects that are observed in this study, which are different from other methods of immobilisation, such as immobilisation on the surfaces of various nanoparticles. In most nanoparticle systems, the observed effects are attributed to an increase of convex curvature, which leads to a minimisation of surface interactions [68], and, for catalysts, an increased accessibility of the active site. The concave curvature of the pores of SBA-15 dramatically increases the surface– protein interaction. Associated stabilisation effects observed by Sang *et al*. were observed as a corresponding increase in protein activity. Also using activity as a gauge for stability, Radhakrishna *et al*. [69] found that immobilisation on the internal surface of SBA-15 increases the activity and stability of alcohol dehydrogenase. Using computational methods it was determined that the elimination of possible open-chain conformations provides entropic effects enhancing the observed increased stabilisation.

Although these results are promising, they merely imply that stabilisation effects are present. To further demonstrate the protection effects, Lynch *et al*. [70] investigated the protection effects rendered by SBA-15 to adsorbed myoglobin in the presence of extreme pH and active protease. Their results directly show that both pH and proteolytic protection effects are present in both high-hydration and high-substrate environments. The results show that confinement decreases the activity of myoglobin at the optimum pH for the free-state protein, although the results also show that confinement induces a broadening in the range of myoglobin activity, and that the resulting activity of the confined myoglobin is much more consistent over the active pH range. This can be seen as a significant advantage of using confined proteins. In practice, the physiological environment in which a therapeutic must operate cannot always be controlled and can also vastly fluctuate [45,46]. The observed broadening and stabilisation of the activity of the confined protein individually addresses each of these issues and allows for a more robust and predictable activity of the resulting therapeutic. This, coupled with the observed proteolytic protection effects, demonstrates the stability and protection effects afforded by the curvature of the SBA-15 system and creates an avenue for therapeutic applications under physiologically challenging conditions.

Apart from pore curvature, other material properties, such as the particle size, mesopore length and particle shape of SBA-15, can also be modulated or tuned to achieve the desired effect. For example, by changing the synthesis process, Zu *et al*. achieved nearly 100% platelet-like particles [59] by varying the duration and stirring speed during micellisation of the P123 template solution. The synthesis of platelet-like particles was optimised for a micellisation time of 1 h with a stirring speed between 800 and 1000 rpm. The particles resulting from this process are ∼ 400 nm long and 1000 nm in diameter. This creates a particle with an inverted aspect ratio as compared with the more common rod-like particles. Also, as the mesopores are aligned along the length of the particle; this morphology reduces the length of the internal mesopores. Depending on the desired application of the particles and how the platelet-like particles can be made, the shortening of the pore length could provide benefits, such as a quicker release of therapeutics at the target site or as another tuning parameter to achieve desired control release dynamics. It should be noted that this method achieves a controlled change in particle morphology without the addition of co-condensates or other structure-directing agents [58,71]. This method leaves the remaining properties of SBA-15 intact and allows for the particle aspect ratio and mesopore length to be varied independently from other therapeutic design parameters. It is also possible to synthesise spherical SBA-15 particles using alternative methods [72]. Similarly to the platelet-like particles, these particles maintain the same properties as the rod-like SBA-15, except that the particles are spherical and range in size from ∼ 3 to 10 µm [72]. These particles also allow for the particle size and mesopore length to be varied independently from other therapeutic design parameters. Prior studies have shown a strong correlation between particle shape and their clearance by phagocytes [25], thus allowing for design of stealth delivery systems or delivery systems that target a specific population of immune cells.

Another design parameter that allows for significant modulation of the particle properties is surface functionalisation. The functionalisation of silica surfaces is well established; this imparts flexibility and reliability on modifying the surface chemistry of SBA-15. In addition, due to the stability of silane bonds, after initial silica functionalisation, well-known organic methods can be used to complete the process using click chemistry [65,73-75]. This ensures the robustness of the surface functionalisation process as any organically conjugated molecule can be used. Further advances in the functionalisation of porous silica, as discussed by Fryxell *et al*. [43], allow for very high-density coverage of functional groups, severely limiting accessibility of any unmodified surface from solution-borne ions. This ensures full control over the surface properties of SBA-15 [43]. A representative example of this method was carried out by Popova *et al*. [65], resulting in a carboxy functionalised surface. This method initially uses 3-amino-propyltriethoxysilane to functionalise the silica surface using silane chemistry with amino groups and then uses succinic anhydride to react with the amine groups in an anhydrous environment to produce carboxyl functional groups. Note that this method follows the principal idea discussed above, that is, the coupling of silane chemistry followed by organic reaction. This study found that the carboxy functionalisation increases the duration of sulfadiazine release without any change of particle *in vitro* cytotoxicity in the Caco-2 cell line. Similarly, Ahmadi *et al*. found that amino functionalisation of SBA-15 significantly increases loading and release duration of ibuprofen [76]. The latter increases from 2 h, releasing only 60% of the loaded therapeutic, to 200 h, releasing 95% of the loaded therapeutic. This kind of behaviour is also seen with low-solubility therapeutics. Mellaerts *et al*. found that high hydroxylation of the SBA-15 surface increases the already enhanced release of the poorly soluble therapeutic itraconazole into a simulated biological fluid [77]. The release creates a supersaturated solution of itraconazole and has allowed formulations for enteral administration to be demonstrated using *in vivo* models [14].

Just as internal surface functionalisation of SBA-15 has been used to control the internal environment of SBA-15, external surface functionalisation is also used to directly address the extra-particle challenge of targeted delivery. This allows for the possibility of limiting therapeutic activity to specific areas and targets such as the blood–brain barrier [78,79], cancerous tissues [64,80], vasculature [81], adipose tissue [82,83], and so on, due to inherent physiological properties and local receptors. Pang *et al*. investigated the targeting of HeLa and A549 cancer cells mediated by the folic acid receptor [64]. This was accomplished by monitoring folic acid conjugated poly(ethylene imine) modified SBA-15 particles loaded with doxorubicin hydrochloride by fluorescence microscopy and flow cytometry. The conjugated particles showed lower cytotoxicity and much greater inhibition of the cancer cells than the non-conjugated particles. The particles also demonstrated sustained release behaviour that is typically desired with this class of therapeutics.

Controlled release of therapeutic cargo can also be regulated by methods other than just the general surface properties, which can yield even greater design flexibility [84,85]. Of these methods, active pore capping [66,86], adaptive polymers [87] and supramolecular assemblies [88] are currently showing great potential. Xue *et al*. [66] have demonstrated controlled release by using lysozyme as a reversible pH responsive nanovalve on mesoporous silica Mobil Composition of Matter-41 (MCM-41). Like SBA-15, MCM-41 is synthesised by macromolecular templating and contains a hexagonally ordered array of mesopores [89]. The template is a cationic, rather than a non-ionic surfactant, namely, cetyltrimethylammonium bromide. The pore walls, however, are thinner and therefore less stable than in SBA-15, and the pore diameter is typically smaller (∼ 1.5 – 10 nm) [90,91]. Controlled release was accomplished even without functionalisation; however, with functionalisation, much more elaborate mechanisms are possible. Xue *et al*. have synthesised organic supramolecule megagates that cap the pores of SBA-15 until exposed to a pH below 5 [88]. These gates have the ability to regulate pores of up to 6.5 nm in diameter and provide yet another tool for SBA-15-based therapeutic development.

Although the insights and tools developed in each of these studies do not separately give way to the next super-drug delivery platform, the nature of the tools and the ability to integrate them leads to many design possibilities. Stability and protection effects from the physiological environment are rendered by the fundamental effects of confinement, curvature and pore surface properties. Targeted delivery and physiological interactions are addressed by flexible external surface functionalisation techniques that allow for a wide range of functional groups. Controlled release is realised by adaptive capping and functionalisation methods that are governed by processes ranging from simple conformational changes to elaborate supramolecular interactions. Figure 2 summarises how the design tools of SBA-15 can be applied to address challenges in the delivery of biological therapeutics. Although this review by no means addresses all of the possible challenges in the delivery of biologics, these tools provide a compelling argument that supports the development of SBA-15 as a serious component in the design of biological therapeutics.

## 6. Expert opinion and future directions

Numerous tools are being developed for the delivery of biological therapeutics. However, many of these tools require the direct modification of the active biologic of interest [92]. This approach can be disadvantageous as it is specific to the active biologic [93] and may require additional information about protein stability and amino acid residues. This approach can also decrease molecular stability, reduce bioactivity, promote undesirable biological interactions including aggregation and association with other proteins, and require significantly different methods of modification for different biological therapeutics. In using SBA-15 as a platform for delivery, the delivery carrier is inherently separated from the active biologic. This makes the platform universal and allows for its application, independently of the desired methods of discovery [94,95] and development [96].

One of the prominent challenges in the delivery of biological therapeutics is that of protein crowding. The difficulties arise at high protein concentrations, for which, in addition to destabilising protein–protein interactions, therapeutic biologics can become too viscous to deliver via injection due to gelation [97]. With targeted delivery, even if the protein concentration in the formulated product is low, the resultant local therapeutic concentration at the target site can still be high. Although this is one of the motivations of targeted delivery that can be used to increase the effectiveness of treatment, it can also lead to complications, which include the instability of the therapeutic. Borwankar *et al*. have been able to prevent gelation at very high protein concentrations through the use of trehalose as a crowding agent [97]. This causes the formation of a stable dispersion of protein nanoclusters that do not gel. Although this addresses protein concentration during delivery, it does not address issues such as targeted delivery and target site behaviour. Alternatively, confinement inside a particle such as SBA-15 does address these issues, in addition to countering gelation at high therapeutic concentrations. The latter is achieved through modulation of the mesopore properties, although targeted delivery and site behaviour is realised by functionalising the external surface of the particle. This allows for a locally high concentration of the therapeutic at the target site without the need for a permanent matrix or the re-engineering of the active biologic [39]. This highlights that, when using the SBA-15 platform, direct modification of the intended therapeutic is not required.

As a consequence, the challenge in implementing the SBA-15 platform lies in the particle design. This revolves around particle biocompatibility and the tunability of both the internal and the external material properties. Although the biocompatibility of this platform is crucial for its success as a therapeutic delivery system, the topic is too broad to be thoroughly considered here. However, three studies are discussed to highlight the work in this area showing the challenges associated with various routes of administration, particle sizes and particle concentrations [98,99]. Hudson *et al*. evaluated the biocompatibility of mesoporous silicas with particle sizes of 150 nm, 800 nm and 4 µm (MCM-41, SBA-15 and mesocellular foam [MCF]) with pore sizes of 3, 7 and 16 nm, respectively. Subcutaneous injection of the materials were well tolerated in a rat model and resulted in elimination of the material progressively over 3 months while showing good biocompatibility on histology. However, the silicates were also shown to have a significant degree of toxicity to mesothelial cells and resulted in death or euthanasia when administered via intraperitoneal or intravenous injection to SV129 mice. On the other hand, Lu *et al*., using a nude mice model, showed that fluorescent mesoporous silica nanoparticles of 130 nm with 2 nm pores are well tolerated via intraperitoneal and intravenous administration [100]. Lu *et al*. have also shown that almost all of the injected particles are excreted via urine and feces within 4 days of administration. Huang *et al*. conducted an *in vivo* study on the effect of mesoporous silica nanoparticles on nude mice xenografted with human malignant melanoma cells (A375) [101]. Surprisingly, the nanoparticles led to an increase in tumour growth. Follow-up *in vitro* studies confirmed that the nanoparticles promoted cellular proliferation and accelerated cell cycle progression of the tumour cells. Furthermore, Huang *et al*. determined that these effects are due to a decreasing of endogenous reactive oxygen species. These three seemingly conflicting studies emphasise contradictory views on the biocompatibility of these materials, and more importantly the fact that the flexibility of the SBA-15 platform is essential for its successful implementation. Size, shape, porosity and surface chemistry are all important, and the ability to control them in SBA-15 is crucial for successful biocompatible design using this platform. This flexibility in design allows for the accurate tuning of many material properties, including surface functionalisation. The difficulty with most current methods is that they impact the functionalisation of the whole, internal and external, surface of SBA-15 particles. This leaves gaps in the literature when selective functionalisation of the internal and external surface is desired. Although methods do exist [102-105], further development in selective functionalisation and the fundamental behaviour of various chemistries would significantly assist in the design of this class of biological therapeutics. Additionally, the inherent design constraints of SBA-15 need to be expanded. Although literature has shown that SBA-15 can be synthesised with pore diameters that are even larger than 30 nm, the larger the pore diameter the less structured the particles become [106]. As many of the beneficial properties of SBA-15 are directly linked to its pore structure and narrow pore size distribution, better methods to synthesise stable, well-structured SBA-15 with larger pore diameters are needed. Also other nanostructured mesoporous materials could be used with different three-dimensional pore topologies, such as SBA-16 [107], FDU-12 [108] and even MCF [109]. However, these may not present the same consistent concave curvature imparted by the cylindrical pores of SBA-15.

Addressing these challenges will enable the rational modulation of both the thermodynamic and kinetic aspects of therapeutic delivery. This can range from designing a strong thermodynamic driving force for protein desorption from the particle, while creating a kinetic barrier to prevent delivery until located at the targeted site (design 1), to optimising the internal pore structure for stability and activity, and the pore size for quick substrate diffusion (design 2).

A possible application of the first hypothetical design would be the delivery of a biological therapeutic across the blood–brain barrier. This could be achieved by the selective functionalisation of the exterior surface of the particle with appropriate ligands and biocompatibility coating. In this case, the ligands could be a ‘marker of self’ peptide resembling CD47 [81,110], and a transferrin recognition peptide [78]. The ‘marker of self’ peptide could act to prevent macrophage-mediated clearance from the vasculature post-injection. This would then allow the transferrin recognition peptide to bind the particle to transferrin and facilitate the particle transfer through the vascular endothelium via receptor-mediated transcytosis. After transport across the blood–brain barrier, the therapeutic could then benefit from enhanced desorption from the particle.

**Figure 3.**
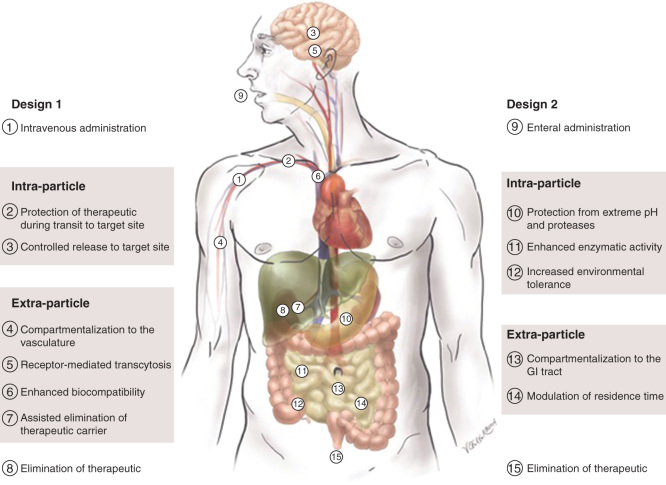
**Overview of potential benefits of discussed therapeutic design cases.** Benefits rendered by the intra- and extra-particle properties of SBA-15 for each of the hypothetical therapeutic delivery designs discussed.

Reproduced with permission ^©^ 2014 Virginia E. Fulford. SBA-15: Santa Barbara Amorphous-15.

In the second design, pore structure and size could be optimised to increase stabilisation of the protein cargo as well as the substrate diffusion rate to the catalytic protein therapeutic. This would increase the effectiveness of the protein used as the catalytic therapeutic. One application could be the confinement of lactase to address lactose intolerance. The stabilisation and protection effects due to confinement would protect the therapeutic when exposed to the extreme environments of the gastrointestinal tract. Particle size and external functionalisation could then prevent the particle from leaving the gastrointestinal tract and select for the desired residence time of the therapeutic particle. An overview of each of these designs can be seen in Figure 3.

Each of these examples illustrates the variability of application without the requirement to modify the intended active therapeutic. This along with the inherent stability effects due to confinement and flexibility in design are the major strengths of the SBA-15 platform.

Article highlights.

- Confinement of biological therapeutics in porous materials enables the inherently distinct intra and extra-particle environments to be leveraged during the therapeutic design process.
- SBA-15 allows independent tuning of key material parameters.
- The SBA-15 platform allows for the delivery of protein therapeutics without direct modification of the desired therapeutic.

This box summarizes key points contained in the article.

## Declaration of interest

The authors gratefully acknowledge the support of the NSF through Grant No. 0333314 and via CBET-0967937, and the EPSRC through a Frontier Engineering Award for the Centre for Nature Inspired Engineering, EP/K038656/1. The authors have no other relevant affiliations or financial involvement with any organization or entity with a financial interest in or financial conflict with the subject matter or materials discussed in the manuscript apart from those disclosed.
